# Do actions speak louder than words? Examining children’s ability to follow instructions

**DOI:** 10.3758/s13421-017-0702-7

**Published:** 2017-03-17

**Authors:** Amanda H. Waterman, Amy L. Atkinson, Sadia S. Aslam, Joni Holmes, Agnieszka Jaroslawska, Richard J. Allen

**Affiliations:** 1 School of Psychology, University of Leeds, Leeds, LS2 9JT UK; 2 MRC Cognition and Brain Sciences Unit, University of Cambridge, Cambridge, UK; 3 School of Psychology, University of Edinburgh, Edinburgh, UK

**Keywords:** Working memory, Learning, Enactment

## Abstract

The ability to encode, retain, and implement instructions within working memory is central to many behaviours, including classroom activities which underpin learning. The three experiments presented here explored how action—planned, enacted, and observed—impacted 6- to 10-year-old’s ability to follow instructions. Experiment 1 (*N* = 81) found enacted recall was superior to verbal recall, but self-enactment at encoding had a negative effect on enacted recall and verbal recall. In contrast, observation of other-enactment (demonstration) at encoding facilitated both types of recall (Experiment 2a: *N* = 81). Further, reducing task demands through a reduced set of possible actions (Experiment 2b; *N* = 64) led to a positive effect of self-enactment at encoding for later recall (both verbal and enacted). Expecting to enact at recall may lead to the creation of an imaginal spatial-motoric plan at encoding that boosts later recall. However, children’s ability to use the additional spatial-motoric codes generated via self-enactment at encoding depends on the demands the task places on central executive resources. Demonstration at encoding appears to reduce executive demands and enable use of these additional forms of coding.

The ability to follow instructions is fundamental to the successful completion of a myriad of behaviours in childhood, including the use of technology, learning to cook, and engagement in leisure activities from board games to sport. In particular, it is a fundamental aspect of a child’s ability to engage effectively in learning activities within the classroom (Engle, Carullo, & Collins, 1991; Gathercole, Lamont, & Alloway, 2006). The capacity to encode, retain, and implement instructed sequences calls upon working memory, both simple working memory (linked to storage—sometimes referred to as short-term memory) and complex working memory (linked to processing and executive control; Baddeley, 2012; Cowan, 2008; Gathercole, Durling, Evans, Jeffcock, & Stone, 2008; Gathercole et al., 2006). Further, many of these instructions involve the engagement of the motoric system via completion of action steps. For example, following a simple recipe, or carrying out sequential instructions within the classroom: ‘Add Compound A to Liquid B, shake the mixture, write down what you observe’. However, the role of action within working memory has been less well studied relative to other modalities (e.g. visual, phonological), and whilst some models have incorporated a movement or motor processing element (e.g. Barrouillet & Camos, 2015; Logie, 1995, 2011), this is not a feature widely explored in most theoretical approaches (Allen & Waterman, 2015; Jaroslawska, Gathercole, Allen, & Holmes, 2016). To date, research has primarily focused on adults’ instruction-following skills. The factors influencing the ability to implement instructions in children are not well understood. Therefore, the three experiments presented here explore the role of action within the working memory framework in children’s ability to follow instructions.

Working memory supports children’s ability to learn (e.g. Gathercole et al., 2016), and working memory skills are linked to children’s educational attainment in core subjects such as reading and mathematics (Gathercole, Brown, & Pickering, 2003; Gathercole, Pickering, Knight, & Stegmann, 2004; Holmes & Adams, 2006; Swanson, Ashbaker, & Lee, 1996). Within the classroom, children are regularly required to remember and implement instructions provided by the teacher, the successful performance of which necessitates the storage of information (simple working memory) and the manipulation of information (complex working memory; Engle et al., 1991; Gathercole et al., 2008). Indeed, Gathercole et al. (2006) observed that children identified as having poor working memory abilities often struggled to follow instructions in class, with resulting detriment to their academic attainment.

In adult participants, an action recall advantage has been demonstrated when implementing instructional sequences within a working memory paradigm (e.g. Allen & Waterman, 2015; Koriat, Ben-Zur, & Nussbaum, 1990; Yang, Allen, & Gathercole, 2015; Yang, Gathercole, & Allen, 2014). For example, Yang et al.’s (2014) participants read a series of action instructions that they then either repeated verbally or acted out. At recall, participants were consistently more accurate when acting out the instructions compared with verbally repeating them. This improved performance with enacted recall suggests that participants make use of spatial-motoric action representations when expecting to enact at recall, which supplements the verbal code generated by spoken instructions (Allen & Waterman, 2015; Brandimonte & Passolunghi, 1994; Freeman & Ellis, 2003; Yang et al., 2014). Within the developmental literature Gathercole et al. (2008) investigated the effect of enactment on children’s recall of instruction sequences within working memory. Five- and 6-year-old children were asked to repeat verbally, or physically to implement, verbally presented instructions using classroom objects (e.g. *pick up the blue ruler and put it in the red box*). The study found that working memory measures were correlated with recall performance (see also Jaroslawska, Gathercole, Logie, & Holmes, 2016), and that children were more accurate when enacting the instructions at recall compared with verbally recalling the instructions, suggesting that children may also benefit from planning to act when listening to verbal instructions.

The motoric system can also be engaged at *encoding*. This was first demonstrated in the long-term memory literature with adults where the effects of enactment at encoding—referred to as self-performed tasks (SPTs)—were investigated on episodic recall (e.g. Engelkamp & Jahn, 2003; Engelkamp & Zimmer, 1989, 1994; Kubik, Obermeyer, Meier, & Knopf, 2014; Nyberg, Nilsson, & Beckman, 1991; Steffens, Jelenec, Mecklenbräuker, & Thompson, 2006). In these studies, participants were asked to memorize long lists of action phrases, with several minutes between encoding and recall, necessitating retrieval from long-term memory. Participants acted out the action phrases (SPT) as they read them (or listened to them) with a control group just reading (or listening). Consistently, these studies found a positive effect of SPT on later verbal recall. In related literature, studies on the use of gestures in learning tasks have found that enactment via gesture is beneficial for performance (e.g. Cutica, Ianì, & Bucciarelli, 2014; Madan & Singhal, 2012). The only study to have explored the effect of SPT with children within the long-term memory paradigm was conducted by Cohen and Stewart (1982). They found children’s verbal recall of action phrases enacted at encoding was better than their verbal recall of words not enacted at encoding. However, the stimuli differed across conditions (the tasks and words were not equivalent), making it hard to draw firm conclusions as to the benefit of SPT for long-term recall in children.

There is some evidence that the motoric system can also be engaged when encoding into working memory (e.g. Smyth & Pendleton, 1989). One small-scale study focusing on children with autism spectrum disorder (Wojcik, Allen, Brown, & Souchay, 2011) found that SPT at encoding provided some benefit to the later recall of instructions. In a more recent study, Jaroslawska, Gathercole, Allen, et al. (2016) investigated the role of enactment at both encoding and recall. Based on the Gathercole et al.’s (2008) methodology, Jaroslawska, Gathercole, Allen, and colleagues (2016) gave 7- to 9-year-olds a series of instructions involving familiar classroom objects (e.g. red pencil, yellow ruler) that contained two different action instructions (i.e. ‘touch’ and ‘pick up’). They orthogonally manipulated enactment at encoding and enactment at recall. Their data showed that enacted recall was better than verbal recall and that enactment at encoding boosted later recall. However, given the familiarity of the objects, and the use of common actions and affordances associated with those objects (e.g. pick up the blue pencil), children may have drawn on knowledge in long-term memory to complete the task, making it hard to derive firm conclusions about the role of enactment within a working memory paradigm. In addition, Jaroslawska, Gathercole, Allen, et al. (2016) did not independently measure children’s working memory abilities and therefore were unable to investigate how different components of working memory (e.g. verbal vs visuospatial; simple vs complex) might relate to task performance in the different conditions.

Within working memory, the distinction is often made between simple working memory tasks that emphasize storage of information (sometimes referred to as short-term memory tasks), and the processing and manipulation of information required by complex working memory tasks (e.g. Baddeley, 1986; Baddeley & Hitch, 1974; Cowan, 2008; Logie, 2011). Abundant evidence suggests that short-term maintenance of information is domain-specific (Baddeley & Hitch, 1974; see Baddeley, 2007, for a review), with verbal (phonological) information stored separately from visuospatial information (e.g. Allen, Havelka, Falcon, Evans, & Darling, 2015; Davis, Rane, & Hiscock, 2013; Logie, Zucco, & Baddeley, 1990; Thalmann & Oberauer, 2016). In addition, these two systems may have distinct developmental trajectories (Gathercole, Pickering, Ambridge, & Wearing, 2004). Understanding how these different proposed components of working memory (e.g. verbal vs visuospatial) might relate to following instructions under verbal and action conditions is something that has not been investigated to date, but it could provide important additional information about the processes underlying task execution.

To summarize, most of the existing literature has focused on the role of motor action on adults’ abilities to follow instructions. A few relevant developmental studies have investigated either enactment at recall *or* at encoding. Only one study by Jaroslawska, Gathercole, Allen, et al. (2016) has combined the two, but this was potentially confounded by long-term memory effects and did not explore the relationship between task performance and performance on working memory measures tapping into different domains. Therefore, it is important to consider how enactment at both encoding and recall relates to task performance in children in a working memory paradigm without these confounds to (i) establish whether the potential benefits of action at both stages reflect a common set of processes, (ii) understand the nature of underlying representations involved in following instructions, and (iii) develop methods to support and improve children’s instruction-following skills in a practical sense.

The experiments presented here used instruction sequences involving novel object-action pairings (based on Allen & Waterman, 2015), which enabled a more robust test of how enactment at encoding and recall affect children’s ability to follow instructions within a working memory paradigm. In addition, a series of standard working memory measures were administered to explore the relationship between different aspects of children’s working memory and instruction following.

## Experiment 1

Improved understanding of the factors affecting children’s ability to follow instructions is important to help maximize learning opportunities in applied contexts, such as the classroom. The current study is based on the methodology used in Allen and Waterman (2015) and explores how enactment at encoding, and enactment at recall, affect children’s following instruction performance. In line with previous research (e.g. Allen & Waterman, 2015; Gathercole et al., 2008; Jaroslawska, Gathercole, Allen, et al., 2016) we would expect children to show an action recall advantage. Based on the Jaroslawska, Gathercole, Allen, et al. (2016) study we might also expect children to show a boost at recall when they have enacted instructions at the encoding phase. However, Jaroslawska, Gathercole, Allen, et al. (2016) used a relatively simple and familiar paradigm. This experiment investigates whether enacted encoding is still facilitative when presented with a more complex set of instructions using novel object-action pairings.

This experiment also explores the link between performance on the different conditions of the following instructions task and different aspects of working memory. Measures of simple verbal/phonological (forward digit recall; FDR), simple visuospatial working memory (Corsi), and complex working memory (backward digit recall; BDR) were taken (see e.g. Pickering & Gathercole, 2001). The benefits of enacted encoding and enacted recall may be linked to the creation of a spatial ‘enactment plan’ (Freeman & Ellis, 2003), either through physical action at encoding, or when *expecting* to perform at later recall. On this basis, performance in the enacted conditions might be linked to visuospatial working memory. The condition involving oral presentation and spoken recall is primarily verbal in nature and does not involve enactment. Performance in this condition might therefore be related to simple verbal working memory as children might automatically engage in sub vocal rehearsal to hold in mind the verbally presented sequences.

### Method

#### Participants

A primary school in West Yorkshire, UK, agreed to participate in this study. There were 81 children aged between 6 and 10 years (mean age = 8 years 4 months, range: 6 years 6 months–10 years 5 months). There were 47 males and 34 females, the children were predominantly White British, and from low to middle SES families. Consent was obtained from parents prior to the research starting, and verbal consent was obtained from the children on the day of testing. Children were told that they did not have to participate and could withdraw at any time. Ethical approval was obtained from the University of Leeds Research Ethics Committee. Consent was obtained from schools (written), parents (written), and individual children (verbal) prior to commencement of the research. This was the case for all three experiments presented in this study.

#### Materials

Sequences of instructions were developed from sets of six actions (flip, lift, push, shake, spin, tap) and six shapes (circle, cross, square, star, sun, triangle). Each sequence consisted of a number of action-object pairs whereby none of the individual actions or objects was repeated within a given sequence (e.g. for a three-pair sequence, *tap the circle, lift the cross, shake the square*). The shapes were presented as black outlines on square laminated cards, each neutral-coloured (white) and measuring 5 cm × 5 cm.

#### Design and procedure

The experiment implemented a 2 × 2 repeated-measures design, with encoding condition (no enactment vs enactment) and recall condition (verbal vs enacted recall) as the independent variables. This resulted in four experimental conditions; no enactment at encoding/verbal recall; enactment at encoding/verbal recall; no enactment at encoding/enacted recall; enactment at encoding/enacted recall. Each of the four experimental conditions was performed in a separate block, in a counterbalanced order across participants, with all four conditions completed in one session.

At the beginning of each session, there was a pretest phase. In this phase the experimenter named each shape and demonstrated each action to familiarize children with the stimuli, and associated actions. Children were then asked to name each shape, and perform each action. This ensured that children were able correctly to identify each shape, and could perform each action in such a way as to be distinguishable from all the other actions. Any child not able successfully to complete this pretest phase would be excluded from the study. However, all children were able to complete this phase successfully.

**Fig. 1:**
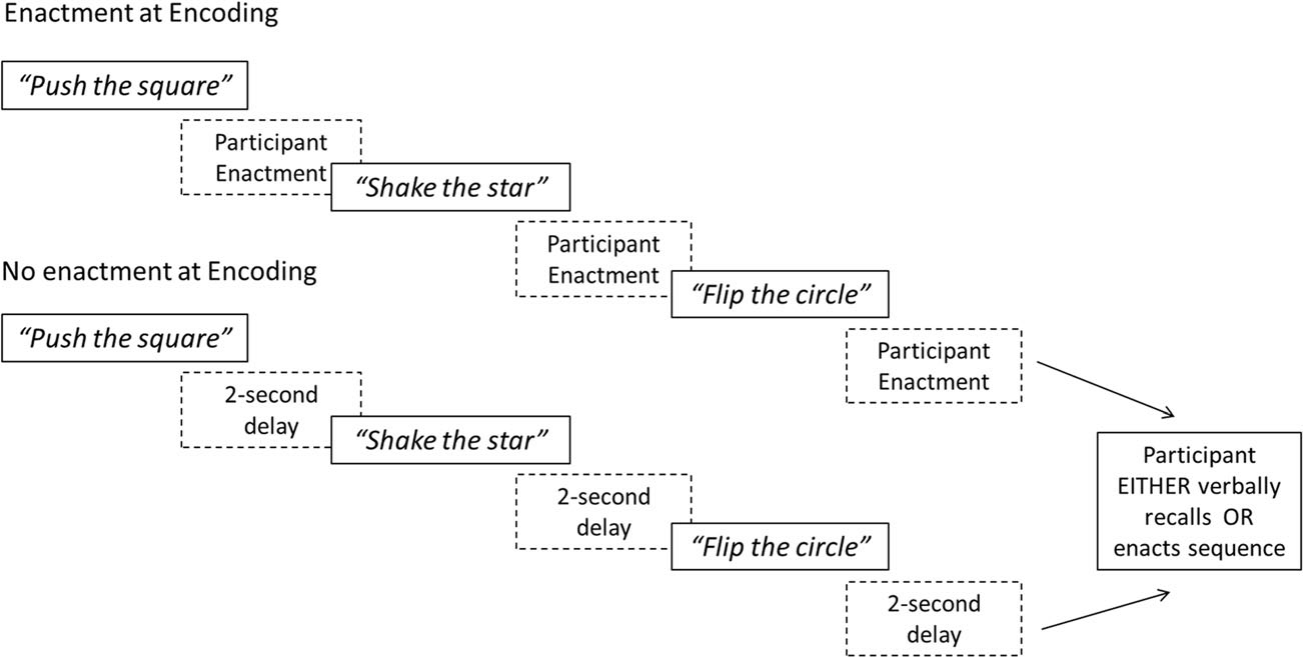
Schematic task diagram showing a sequence of three action-object pairs

For the test phase (see Fig. 1), shapes were placed on the table in front of the participant, in a pseudorandom spatial configuration that remained constant for each participant. Each experimental condition began with two practice trials containing one action-object pair, and two practice trials involving two pairs, before proceeding to the experimental trials. For the experimental trials all participants performed five sequences containing two object-action pairs (e.g. *flip the circle, lift the cross*), followed by five sequences containing three object-action pairs (e.g. *push the square, shake the star, spin the sun*), and five sequences containing four object-action pairs (e.g. *tap the triangle, flip the sun, lift the star, spin the circle*). (See Appendix 1 for instructions given to children in the test phase.)

#### Encoding

Both encoding conditions involved verbal presentation of the sequence by the experimenter. For the enactment condition, participants performed each action-object pair during the interstimulus interval, with the object immediately placed back in its original position by the experimenter following this movement.^1^ For the no enactment condition, participants simply listened to the instructions. In order to match the time between presentations of each action-object pair across the two encoding conditions, there was a pause of two seconds after the verbal presentation of each action-object pair. This accounted for the extra time taken for children to perform the instruction in this inter-stimulus interval in the enactment condition. Shapes remained visible throughout all phases of all conditions. Children were told to remain silent during encoding, and children in the verbal encoding condition were told not to touch the objects during encoding.

#### Recall

This immediately followed the final 2-second delay of encoding for each sequence. For the verbal recall condition, participants had to verbally recall the entire set of action-object pairs, in their original serial order. For the enacted recall condition participants had to physically carry out each of the action-object instructions in turn, in exactly the same order in which they were presented. Children in the verbal recall condition were told not to touch the objects during recall, and children in the enacted recall condition were told not to speak during recall.

In addition, children were tested on three standard working memory measures: FDR, Corsi and BDR (see e.g. Alloway, 2007; Alloway, Gathercole, & Pickering, 2006; Gathercole, 1999; Vandierendonck, Kemps, Fastame, & Szmalec, 2004). For FDR, a series of digits was read aloud by the experimenter, and the participant had to repeat the digits in the order in which they were presented (e.g. if the experimenter said ‘3, 7, 2’, the participant had to repeat ‘3, 7, 2’). For BDR, the procedure was the same except participants had to repeat the digits in *reverse* order. For the Corsi task, a board was placed on the table in front of the participant on which there were nine randomly spaced ‘blocks’. The experimenter tapped out spatial sequences on the blocks and participants had to repeat the spatial pattern immediately following presentation. For each task, a span methodology was used where the number of to-be-remembered items increased from length two to length eight. There were three sequences at each length. If participants got two out of the three sequences correct at a given length, they moved onto the next length. If they made errors on two (or more) of the sequences at a given length, the test was stopped. The participant’s score was the last length at which they correctly remembered at least two out of the three sequences. If the participants correctly remembered one of the three sequences at the next length (rather than zero out of three), an additional 0.33 was added to their score.

### Results

#### Following instructions

**Fig. 2:**
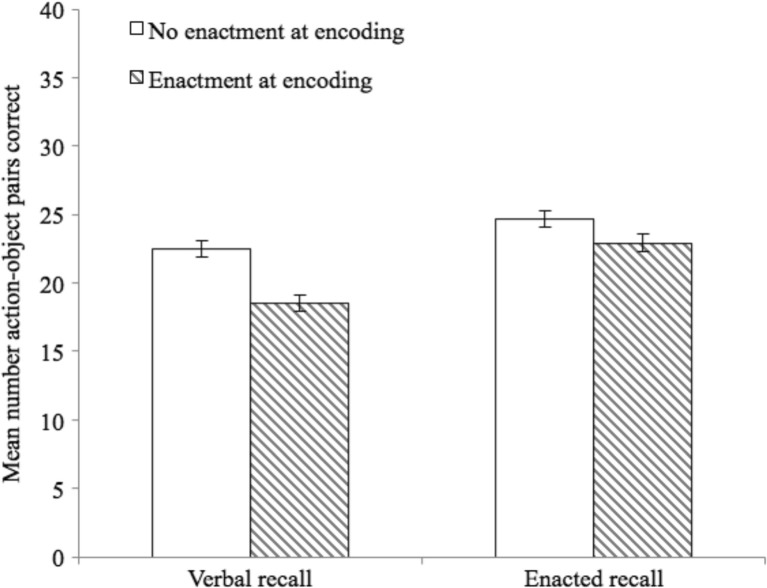
Mean number of action-object pairs correctly recalled (with standard error) for Experiment 1

The dependent variable was the total number of action-object pairs correctly recalled in each condition. A 2 (encoding condition) × 2 (recall condition) repeated-measures ANOVA was run.^2^ There was a significant main effect of recall, *F*(1,78) = 86.50, *p* < .001, η_p_^2^ = .53, with enacted recall (*M* = 21.43; 95% CI [20.32, 22.54]) superior to verbal recall (*M* = 17.36; 95% CI [16.28, 18.45]). The effect of encoding was also significant, *F*(1,78) = 8.30, *p* < .01, η_p_^2^ = .10, with children performing better with no enactment at encoding (*M* = 20.15; 95% CI [19.04, 21.24]), relative to enactment at encoding (*M* = 18.64; 95% CI [17.48, 19.80]). This was qualified by an interaction between encoding and recall, *F*(1,78) = 4.82, *p* < .05, η_p_^2^ = .06 (see Fig. 2). Planned comparisons showed that for enactment at encoding, enacted recall was better than verbal recall, *t*(80) = -8.05, *p* < .001, Cohen’s *d* = -0.81. For no enactment at encoding, enacted recall was still better but with a reduced effect size, *t*(80) = -5.77, *p* < .001, *d* = 0.56. For verbal recall, performance was less accurate following enactment at encoding, relative to no enactment, *t*(80) = 4.14, *p* < .001, *d* = 0.42, while the difference between these encoding conditions for enacted recall was not significant, [*t*(80) = .90, *p* > .05, *d* = .10.

#### Working memory measures

Partial correlations (controlling for age) were run on performance across the four conditions of the Following Instructions task, and scores on the three working memory measures (FDR, BDR, Corsi; see Table 1). Working memory measures that significantly correlated with task performance were then entered into a hierarchical regression analysis for each of the four conditions. Age (in months) was always entered in the first step, then the relevant working memory measures were entered in the second step. For no enactment at encoding/verbal recall, the model at Step 1 was significant, *F*(1, 80) = 11.21, *p* < .01. The model at Step 2 made a significant additional contribution (∆*R* ^*2*^ = .31, *p* < .001), with FDR a unique predictor of task performance, β = .49, *t*(80) = 4.89, *p* < .001. For no enactment at encoding/enacted recall, the model at Step 1 was significant, *F*(1, 80) = 8.13, *p* < .01. The model at Step 2 made a significant additional contribution (∆*R* ^2^ = .27, *p* < .001), with all three working memory measures uniquely predicting task performance (although note that Corsi was only marginally significant at *p* = .058), FDR: β = .28, *t*(80) = 2.58, *p* < .05; BDR: β = .29, *t*(80) = 2.69, *p* < .05; Corsi: β = .20, *t*(80) = 1.92, *p* = .058. For enactment at encoding/verbal recall the model at Step 1 was significant, *F*(1, 80) = 9.83, *p* < .01. The model at Step 2 made a significant additional contribution (∆*R* ^2^ = .12, *p* < .01), with BDR uniquely predicting task performance, β = .28, *t*(80) = 2.36, *p* < .05. Finally, for enactment at encoding/enacted recall the model at Step 1 was significant, *F*(1, 80) = 12.91, *p* < .01. The model at Step 2 made a significant additional contribution (∆*R* ^2^ = .09, *p* < .05), with BDR uniquely predicting task performance, β = .26, *t*(80) = 2.19, *p* < .05.

**Table 1:** Partial correlation analysis (controlling for age) for measures of WM and following instructions (as a function of experimental condition) in Experiment 1

	No enactment encoding/Verbal recall	No enactment encoding/Enacted recall	Enactment encoding/ Verbal recall	Enactment encoding/ Enacted recall
FDR	.58***	.44***	.27*	.18
BDR	.40***	.48***	.42***	.36**
Corsi	.22*	.33**	.16	.17

*Note.* FDR = forward digit recall; BDR = backward digit recall. * *p* < .05. ** *p* < .01. *** *p* < .001

### Discussion

In line with previous research (e.g. Gathercole et al., 2008; Jaroslawska, Gathercole, Allen, et al., 2016) there was a robust effect of action at recall, with enacted recall superior to verbal recall. Allen and Waterman (2015) argue that the enacted recall advantage in adults reflects the creation of an imaginal spatial-motoric plan when expecting to enact at recall (see also Freeman & Ellis, 2003). This study supports the idea that children can also take advantage of this additional spatial-motoric plan when expecting to enact. Indeed, the relationship observed between children’s visuospatial working memory abilities and their performance in the condition where they are listening to instructions that they are expecting to enact at recall suggests a role for visuomotor planning. These results could also be considered from a ‘levels-of-processing’ point of view (Craik & Lockhart, 1972; Craik & Tulving, 1975). For the entirely verbal condition (with no enactment at either encoding or recall), children could potentially perform the task with only a superficial level of encoding (e.g. subvocal rehearsal). For any of the three conditions involving enactment, children would potentially have to represent the instructions’ meaning via semantic processing, involving a deeper level of processing. This would support the beneficial effect of enacted recall, but does not easily explain why performance on the condition where children enact at encoding then recall verbally, is worse than the verbal only condition.

In contrast to Jaroslawska, Gathercole, Allen, et al. (2016), enactment during encoding had a negative effect on children’s memory for instruction sequences, irrespective of the type of recall required (verbal or enacted). The instructions task used here involved novel object-action pairs (e.g. *shake the triangle*) and a more complex set of actions than the potentially more familiar pairings (e.g. pick up the blue pencil) used by Jaroslawska, Gathercole, Allen, et al. (2016). One possibility is that the current, more complex, task placed heavier demands on children’s working memory resources. This extra load on working memory might then prevent children from using extra spatial-motoric codes available via enactment at encoding to benefit recall performance. The lack of a relationship between visuospatial working memory and conditions involving action at encoding supports this interpretation.

Instruction following was supported by complex working memory in all conditions except the purely verbal condition. This likely reflects the executive demands of simultaneously holding in mind and either planning for or performing the steps of the instructions. Performance in the purely verbal condition was underpinned by simple verbal working memory only. Given that children simply had to listen to verbal instructions and then repeat them verbally in the order they were presented, we would expect that verbal short-term memory would be the key component in successfully completing this task. Verbal short-term memory also supported performance in all conditions except enacted encoding/enacted recall. This suggests that children may automatically engage in subvocal rehearsal to maintain verbally presented instructions, except when there is a strong motor component to the task.

Based on the findings of the current experiments, enactment at encoding does not appear to provide a useful method of boosting children’s recall of instructions, at least in the current paradigm.^3^ This suggests that the effect of enactment at encoding is task dependent, and therefore conclusions about the efficacy (or not) of enacted encoding need to be moderated by an analysis of the task requirements. To explore this idea further, two additional experiments were run to examine different ways to reduce task demands to assess whether this would alter the effect of enactment at encoding. Experiment 2a investigates the impact of observing demonstration of instructions at encoding, and therefore potentially providing the extra spatial-motoric code but without the possible costs associated with self-enactment at encoding. Experiment 2b reduced the number of possible actions to two, in line with the Jaroslawska, Gathercole, Allen, et al. (2016) study.

## Experiment 2a

Research examining the effects of observing demonstration of instructions may help shed light on the role of enactment at encoding when following instructions. In the adult literature, Yang et al. (2015) found a small benefit for demonstration at encoding delivered via a computer screen for adults’ subsequent recall within working memory. Similarly, in a small study focusing on children with ASD, Wojcik et al. (2011) showed that experimenter demonstration at encoding benefitted recall. In addition, studies using long-term memory paradigms have shown that experimenter-performed tasks (EPT) during encoding improved adults’ long-term free recall and cued recall (e.g. Engelkamp & Dehn, 2000; Steffens, 2007). This also links to the literature on mirror neurons, whereby certain neurons are active both when observing and executing similar goal-directed actions (Kilner, Neal, Weiskopf, Friston, & Frith, 2009; Nelissen, Luppino, Vanduffel, Rizzolatti, & Orban, 2005; Rizzolatti, Fadiga, Gallese, & Fogassi, 1996), suggesting a shared neuronal substrate for motoric performance and observation of motor acts.

Enacted demonstration of to-be-remembered instructional sequences may therefore provide additional spatial-motoric representational codes to support performance, without requiring physical enactment at the encoding stage. If it is the requirement to produce actual motoric responses during encoding that produces the negative effect on children’s following instruction performance, then EPT may serve to boost performance on the following instructions task. This experiment therefore examined whether recall for sequences of instructions is improved when their verbal presentation is supplemented by additional experimenter demonstration during encoding. As with Experiment 1, measures were taken of children’s simple (verbal and spatial) and complex working memory ability. If experimenter demonstration at encoding enables children to make use of spatial-motoric information at encoding, then children’s performance on the visuospatial working memory task (Corsi) might predict unique variance in their recall performance on the following instructions task in the experimenter-demonstration conditions.

### Method

#### Participants

A second primary school in West Yorkshire, UK, agreed to participate in this study. None of the children in this experiment had taken part in Experiment 1. There were 81 children between 6 and 10 years of age (mean age = 8 years 4 months; range: 6 years 7 months–10 years 4 months). There were 41 males and 40 females, the children were predominantly White British, and from low to middle SES.

#### Design, materials, and procedure

This was identical to Experiment 1, including the materials, and the pretest phase to familiarize children with the task. The only difference was during the encoding phase of the experimental conditions. In Experiment 2a, instead of enactment at encoding versus no enactment at encoding, the experimental manipulation was demonstration at encoding versus no demonstration at encoding (note that the no enactment at encoding condition of Experiment 1 and the no demonstration at encoding condition in Experiment 2a are identical). As with Experiment 1, in order to match the task across the encoding conditions (i.e. to account for the time taken for experimenter demonstration in the demonstration condition), there was a 2-second interstimulus interval in both encoding conditions. Therefore, in the demonstration condition children watched the experimenter act out each of the to-be-remembered instructional elements in the interval between verbal presentation of each action-object pair.

### Results

#### Following instructions

**Fig. 3:**
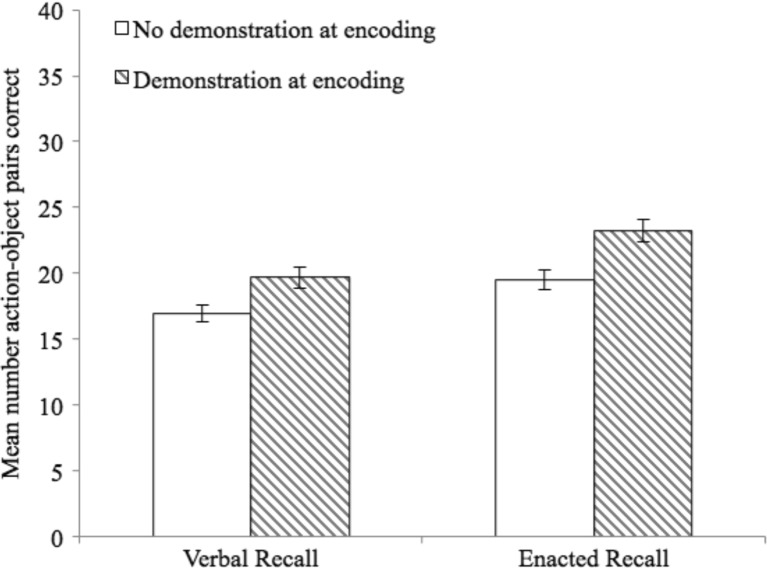
Mean number of action-object pairs correctly recalled (with standard error) for Experiment 2a

The dependent variable was the total number of action-object pairs correctly recalled in each condition. A 2 (encoding) × 2 (recall) repeated-measures ANOVA showed a significant main effect of recall, *F*(1, 78) = 43.29, *p* < .001, η_p_^2^ = .36, with enacted recall (*M* = 21.30; 95% CI [20.03, 22.57]) superior to verbal recall (*M* = 18.25; 95% CI [17.00, 19.51]). The effect of encoding was also significant, *F*(1,78) = 52.00, *p* < .001, η_p_^2^ = .40, with children performing better in the demonstration conditions (*M* = 21.37; 95% CI [20.02, 22.72]) than no demonstration (*M* = 18.18; 95% CI [17.03, 19.33]; see Fig. 3). The interaction was not significant (*F* < 1).^4^

#### Working memory measures

Partial correlations (see Table 2) and regression analyses were run as per Experiment 1. For no demonstration at encoding/verbal recall, the model at Step 1 was significant, *F*(1, 80) = 4.12, *p* < .05. The model at Step 2 made a significant additional contribution (∆*R* ^2^ = .38, *p* < .001), with FDR a unique predictor of task performance, β = .59, *t*(80) = 6.57, *p* < .001. For no demonstration at encoding/enacted recall, the model at Step 1 was significant, *F*(1, 80) = 24.83, *p* < .001. The model at Step 2 made a significant additional contribution (∆*R* ^2^ = .27, *p* < .001), with FDR: β = .43, *t*(80) = 5.12, *p* < .001, and Corsi: β = .19, *t*(80) = 2.14, *p* < .05, uniquely predicting task performance. For demonstration at encoding/verbal recall the model at Step 1 was significant, *F*(1, 80) = 10.18, *p* < .01. The model at Step 2 made a significant additional contribution (∆ *R* ^2^ = .35, *p* < .001), with FDR: β = .51, *t*(80) = 5.85, *p* < .001, and Corsi: β = .26, *t*(80) = 2.74, *p* < .01, uniquely predicting task performance. Finally, for demonstration at encoding/enacted recall the model at Step 1 was significant, *F*(1, 80) = 18.12, *p* < .001. The model at Step 2 made a significant additional contribution (∆ *R* ^2^ = .17, *p* < .001), with FDR: β = .34, *t*(80) = 3.57, *p* < .01, and Corsi: β = .19, *t*(80) = 1.99, *p* < .05, uniquely predicting task performance.

**Table 2:** Partial correlation analysis (controlling for age) for measures of WM and following instructions (as a function of experimental condition) in Experiment 2a

	No Demo encoding/Verbal recall	No Demo encoding/Enacted recall	Demo encoding/ Verbal recall	Demo encoding/ Enacted recall
FDR	.63***	.54***	.67***	.41***
BDR	.21	.27*	.16	.17
Corsi	.16	.27*	.32**	.24*

*Note.* FDR = forward digit recall; BDR = backward digit recall. * *p* < .05. ** *p* < .01. *** *p* < .001

### Discussion

In contrast to Experiment 1, action at encoding had a positive effect across both recall conditions. Thus, children’s observation of experimenter enactment for to-be-remembered instructions does not appear to produce the same ‘overloading’ of working memory as found with self-enactment during encoding. Taken together, these findings suggest that the *mental* representation of enactment at encoding (either derived through observation of enactment by others, or planning for enactment by the participant) appears to be beneficial, whereas actual physical enactment during instruction has negative effects in children. In line with this, visuospatial working memory resources were recruited for the maintenance of demonstrated enactment in the current study but not for self-enactment in Experiment 1. The role of complex working memory also differs across studies with a greater reliance on executive resources under self-enactment conditions (Experiment 1) than experimenter enactment (Experiment 2a). These findings suggest that visual demonstration places fewer demands on executive control in comparison to those placed by self-enactment and that children are able to benefit from the use of additional spatial-motoric codes when they are not engaged in effortful *self*-enactment. Once again, because the task requires listening to verbal instructions, simple verbal working memory played an important role across conditions.

The current study showed that children can benefit from additional spatial-motoric codes at encoding when demands are reduced through observation of other-enactment. However, Jaroslawska, Gathercole, Allen, et al. (2016) showed that self-enactment at encoding is not always detrimental to recall and that self-enactment at encoding can boost memory performance. One difference between the two studies is the number of possible actions required to complete the instructions. The current methodology draws from a set of six possible actions, whereas Jaroslawska, Gathercole, Allen, et al. (2016) only used two possible actions. The more limited number of possible actions may serve to reduce task demands and lead to different results for enacted encoding. Therefore, Experiment 2b explored whether simply reducing the number of possible actions in the current methodology (from six to two) would alter the effect of self-enactment at encoding.

## Experiment 2b

Exploring how task demands affect the interaction between enactment at encoding and recall of instructions will shed further light on the possible processes underlying children’s ability to follow instructions. Indeed, with regards to the applied aspects of this work (e.g. in relation to classroom activities), it is important to understand under what conditions enactment could help boost children’s performance.

### Method

#### Participants

A third primary school in West Yorkshire, UK, agreed to participate in this study. None of the children in this experiment had taken part in either Experiment 1 or Experiment 2a. There were 64 children aged between 6 and 10 years (mean age = 8 years 4 months; range: 6 years 4 months–10 years 4 months). There were 32 males and 32 females, the children were predominantly White British and from low to middle SES.

#### Design, materials, and procedure

This was identical to Experiment 1, including the materials, and the pretest phase to familiarize children with the task. The only difference was that the possible number of actions used to create the object/action instruction pairs was reduced from six to two (‘push’ and ‘lift’).

### Results

#### Following instructions

**Fig. 4:**
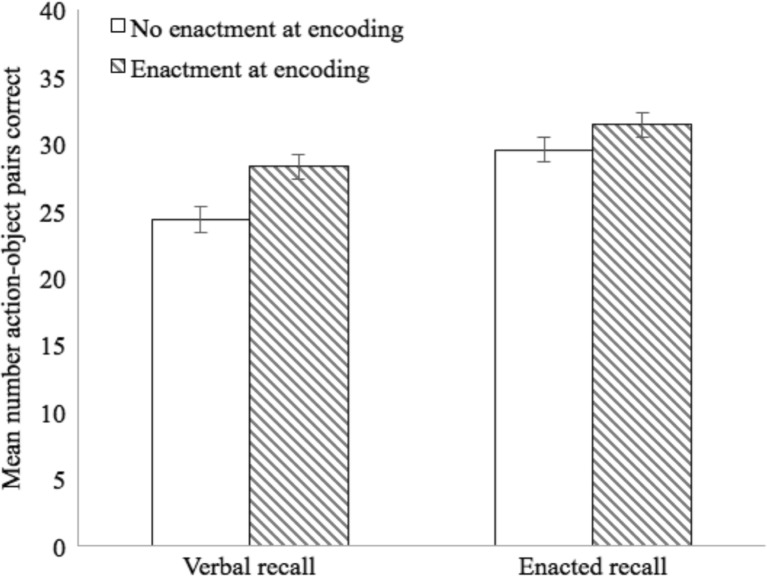
Mean number of action-object pairs correctly recalled (with standard error) for Experiment 2b

The dependent variable was the total number of action-object pairs correctly recalled in each condition. A 2 (encoding) × 2 (recall) repeated-measures ANOVA showed a significant main effect of recall, *F*(1, 63) = 42.52, *p* < .001, η_p_^2^ = .40, with enacted recall (*M* = 29.86; 95% CI [28.10, 31.61]) superior to verbal recall (*M* = 26.96; 95% CI [25.34, 28.59]). The effect of encoding was also significant, *F*(1, 63) = 42.02, *p* < .001, η_p_^2^ = .40, with enactment at encoding (*M* = 30.50; 95% CI [28.76, 32.24]) superior to no enactment at encoding (*M* = 26.32; 95% CI [24.55, 28.09]; see Fig. 4). This was qualified by an interaction between recall and encoding, *F*(1,63) = 4.67, *p* < .05, η_p_^2^ = .07. Planned comparisons showed that enactment at encoding boosted both verbal and enacted recall, but the effect was greater for verbal recall, *t*(63) = 5.66, *p* < .001, Cohen’s *d* = 0.71, compared with enacted recall, *t*(63) = 4.77, *p* < .001, *d* = 0.60. Comparing the two recall conditions indicated an advantage for enacted over verbal recall for both no enactment at encoding, *t*(63) = 5.48, *p* < .001, *d* = 0.51, and enactment at encoding, *t*(63) = 3.25, *p* < .001, *d* = 0.26, though this effect was smaller in the latter case.

#### Working memory measures

Partial correlations (see Table 3) and regression analyses were run as per Experiment 1. For no enactment at encoding/verbal recall, the model at Step 1 was significant, *F*(1, 80) = 20.04, *p* < .001. The model at Step 2 made a significant additional contribution (∆ *R* ^2^ = .22, *p* < .001), with FDR a unique predictor of task performance, β = .42, *t*(80) = 4.23, *p* < .001. For no enactment at encoding/enacted recall, the model at Step 1 was significant, *F*(1, 80) = 51.16, *p* < .001. The model at Step 2 made a significant additional contribution (∆*R* ^2^ = .17, *p* < .001), with FDR: β = .26, *t*(80) = 3.04, *p* < .01, and Corsi: β = .28, *t*(80) = 2.71, *p* < .01, uniquely predicting task performance. For enactment at encoding/verbal recall the model at Step 1 was significant, *F*(1, 80) = 34.91, *p* < .001. The model at Step 2 made a significant additional contribution (∆*R* ^2^ = .28, *p* < .001), with BDR: β = .39, *t*(80) = 4.44, *p* < .001, and Corsi: β = .37, *t*(80) = 3.72, *p* < .001, uniquely predicting task performance. Finally, for enactment at encoding/enacted recall the model at Step 1 was significant, *F*(1, 80) = 37.16, *p* < .001. The model at Step 2 made a significant additional contribution (∆*R* ^2^ = .24, *p* < .001), with FDR: β = .17, *t*(80) = 2.04, *p* < .05, BDR: β = .36, *t*(80) = 3.98, *p* < .001, and Corsi: β = .31, *t*(80) = 2.97, *p* < .01, all uniquely predicting task performance.

**Table 3:** Partial correlation analysis (controlling for age) for measures of WM and following instructions (as a function of experimental condition) in Experiment 2b

	No enactment encoding/Verbal recall	No enactment encoding/Enacted recall	Enactment encoding/ Verbal recall	Enactment encoding/ Enacted recall
FDR	.50***	.38**	.23	.29*
BDR	.25*	.32*	.53***	.50**
Corsi	.13	.36**	.46***	.39**

*Note.* FDR = forward digit recall; BDR = backward digit recall. * *p* < .05. ** *p* < .01. *** *p* < .001

### Discussion

As with Experiments 1 and 2a, the enacted recall advantage was replicated. Of more interest is the finding with regards to (self) enactment at encoding. By reducing the number of possible actions from six to two, and thereby reducing the demands of the task, we now observe a facilitative effect on later recall. In addition, whilst enactment at encoding boosted both verbal and enacted recall, it had a greater positive effect on the former. This is in line with research with adults by Allen and Waterman (2015), and with children by Jaroslawka, Gathercole, Allen, et al. (2016). If participants are already expecting to enact at recall, they appear to form an imaginal spatial-motoric plan which is not substantially further supported by actual enactment at encoding. In contrast, if participants are expecting simply to repeat instructions verbally at recall, they may not construct this type of spatial-motoric code unless explicitly required to do so via instructions to enact during encoding (Allen & Waterman, 2015).

As with Experiment 2a, the benefits of enactment at encoding were supported by visuospatial aspects of working memory. This supports the idea that when enactment at encoding is beneficial (rather than detrimental) it is because children are able to utilize additional spatial-motoric codes. However, in contrast to Experiment 2a, complex working memory also contributed to the performance in the enacted-encoding conditions. This may reflect the fact that, whilst children were benefitting from self-enactment at encoding, the process of self-enactment is still relatively demanding and requires the involvement of executive processes. This is in comparison to observing other enactment which appeared to place fewer demands on executive control in Experiment 2a.

## General discussion

The results presented here from three experiments provide insights into children’s ability to follow instructions, and the role of ‘action’ within the working memory framework. Specifically, the data replicate and extend the enacted recall advantage (e.g. Gathercole et al., 2008; Jaroslawska, Gathercole, Allen, et al., 2016). When expecting to enact at recall, children appear to create a plan for enactment that supplements the verbal code with spatial-motoric codes (Allen & Waterman, 2015; Freeman & Ellis, 2003). However, the effect of enactment at *encoding* was shown to depend on task demands. Using novel object-action pairings, rather than the more familiar pairings employed by Jaroslawska, Gathercole, Allen, et al. (2016), a negative effect of self-enactment at encoding was found in Experiment 1. Children were unable to use the additional spatial-motoric codes potentially available from physically acting out instructions prior to recall. Experiments 2a and 2b showed that reducing task demands, either through observed other enactment or by reducing the number of possible actions required with self-enactment, enabled children to make use of these additional codes and led to improved recall. This was supported by exploring the role of working memory in task performance. Visuospatial working memory was linked to task performance when enactment boosted recall, but not when enactment hindered recall. This further extends previous research by enabling a more thorough exploration of the potential underlying mechanisms by which action affects children’s ability to follow instructions within a working memory paradigm.

Interpretation of our findings has been couched primarily within the working memory paradigm, in line with the focus of the present study and the nature of the tasks implemented. Thus, adopting and extending the multicomponent working memory model (Baddeley, 2012), verbal and visuospatial processing may be supported by phonological and visuospatial subcomponents, respectively, while motoric information may be retained in a specialized motor store (Allen & Waterman, 2015; Jaroslawska, Gathercole, Allen, et al., 2016; Smyth & Pendleton, 1989). This is also consistent with an embodied approach to cognition, whereby resources responsible for perceptual-motor processing are able to contribute to temporary storage in tasks designed to measure working memory (Wilson, 2001, 2002). Macken and colleagues (e.g., Macken, Taylor, & Jones, 2015; Macken, Taylor, Kozlov, Hughes, & Jones, 2016) have also recently conceptualized short-term memory paradigms as representing perceptual-motor task sets, although they reject the notion of limited capacity short-term memory systems per se. Nevertheless, Macken and colleagues provide a potentially useful framework within which to interpret the effects of action on following instructions observed in the present study. They argue for the importance of considering the dynamic interplay between three aspects of any experimental context: the task (the process that needs to be completed), the repertoire (perceptuomotor and cognitive abilities of the participant), and the material (the form within which the task is completed). Experiment 1 found a negative effect of enactment at encoding on children’s later recall; this was in contrast to Allen and Waterman (2015) who found a positive effect with adult participants using the same methodology. Within Macken et al.’s (2015) framework, this would equate to differences in repertoire where children’s more limited working memory resources means they are unable to use the additional spatial-motoric codes provided by self-enactment. In addition, the negative effect of enactment at encoding observed in Experiment 1 was in contrast to the positive effects found in Experiments 2a and 2b, and in Jaroslawka, Gathercole, Allen, et al. (2016). This might be explained by differences in materials, either through a simplified action set or the provision of visuospatial information via demonstration. Finally, the consistently positive effect of enacted recall (compared to verbal recall) across all studies may represent differences in the nature of the task, given the different processes required to enact instructions compared to verbally repeating them. Further research that systematically varies each of these three aspects would be interesting in order to explore more fully the differing effects of task, materials, and repertoire on performance.

Alternatively, some researchers have argued for working memory to be conceptualized as activated long-term memory (Cowan, 1995; Nairne, 2002; Ruchkin, Grafman, Cameron, & Berndt, 2003). Such theoretical approaches describe a unitary memory system, supported by research showing similar effects of certain manipulations in both working memory and long-term memory tasks (Brown, Neath, & Chater, 2007; Crowder, 1993). In these models (e.g. Cowan, 1995), the role of attention is important in maintaining and limiting activation of long-term memory. Within the self-enactment paradigm, performing an action could be seen to increase the likelihood of attending to the instruction, compared to passive listening, or, indeed, it could serve as a form of elaborative encoding. However, the logical outcome of this would be for self-enactment always to improve instruction recall, which is not the case. Recent research using an individual differences perspective has suggested that working memory and long-term memory have both shared and unique processes (Unsworth, 2010), and further work in the following instructions paradigm could help to understand the nature of these similarities and differences.

Whilst we assume that the introduction of action-based manipulations in this paradigm supports development of spatial-motoric representational codes that potentially facilitate performance, it is also possible that children additionally engage in active rehearsal strategies (e.g. subvocal or mental rehearsal) that might differ across conditions. Future work might therefore apply dual-task methodology, for example, to explore whether children engage in mental rehearsal of the motoric sequence during encoding. Children could be asked to repeat a simple hand or finger movement during the encoding phase, to disrupt possible mental motoric rehearsal (see e.g. Gimenes, Pennequin, & Mercer, 2016, for similar work in adults). If performance declined in comparison to a condition with no motoric interference during encoding, this would provide evidence that children are using mental rehearsal.

Regarding the application of these data to more applied contexts, the results presented here show the importance of task context in making recommendations. It is clear from the three experiments presented here, and previous research, that children benefit from actively listening to instructions that are to be physically implemented. With regard to enacted encoding, this may prove beneficial for simple tasks, but as task demands increase the positive effect of self-enactment may disappear and instead prove detrimental to recalling instructions. In contrast, physical demonstration may be a more useful technique to apply: observation of other enactment had a beneficial effect even with the more demanding action set used in Experiment 1. The potentially low-cost and nonstrategic nature of this benefit means it might be usefully applied across all abilities and age groups, including children with working memory problems who may otherwise struggle with following instructions (Alloway & Gathercole, 2006). In addition, further research extending to an older adult population would be useful to investigate how the role of action in working memory changes over the lifespan. Following instructions has important practical implications in older adulthood, such as adhering to medication schedules (Marek & Antle, 2008), as well as learning new skills to facilitate engagement with advances in technology. Finally, research which seeks to examine the effect of enactment at encoding on working memory *and* long-term memory within the same task would be interesting to better understand potential differences in how enactment at encoding affects short-term and long-term recall.

To sum, following instructions is a fundamental aspect of a range of everyday behaviours. The results presented here suggest that children benefit from the creation of additional visuospatial and motoric codes when expecting to physically enact at recall. In contrast, actual enactment at encoding depends on the task demands. The potential negative effect of action at encoding can be reversed by reducing executive control demands through observation of enactment rather than self-enactment, with resultant implications for helping children to learn effectively in the classroom.

## Appendix 1: Instructions for test phase

### Experiment 1 and Experiment 2b

#### No enactment at encoding/verbal recall

Now I am going to read out the instructions and you have to listen to what I say. When I have finished reading out the instructions I want you to tell me everything I just said, in the same order. So if I read out ‘lift the square, push the circle’, you would listen to what I say, then you would say ‘lift the square, push the circle’.

We’re going to do some practicing first, so you can get used to what you need to do. In the practice, we will start with one thing and then there will be more for you to remember each time.

[Children are given practice sequences]

Ok, we have finished the practice ones, and now we’re going to start. The number of things I read out gets longer, so you have to listen very carefully.

[Children are given test sequences]

#### No enactment at encoding/enacted recall

Now I am going to read out the instructions and you have to listen to what I say. When I have finished reading out the instructions I want you to act out everything I just said, in the same order. So if I read out ‘lift the square, push the circle’, you would listen to what I say, then you would act out ‘lift the square, push the circle’.

We’re going to do some practicing first, so you can get used to what you need to do. In the practice, we will start with one thing and then there will be more for you to remember each time.

[Practice and test procedure then proceeds as above, for all other conditions]

#### Enactment at encoding/verbal recall

Now I am going to read out the instructions, and you have to act out each instruction as I say it. When I have finished reading out the instructions I want you to tell me everything I just said, in the same order. So if I read out ‘lift the square, push the circle’, you would act out ‘lift the square’ as soon as I say it, then you would act out ‘push the circle’ as soon as I say it. When I’ve finished reading the instructions, you would then say ‘lift the square, push the circle’. We’re going to do some practicing first, so you can get used to what you need to do. In the practice, we will start with one thing and then there will be more for you to remember each time.

#### Enactment at encoding/enacted recall

Now I am going to read out the instructions, and you have to act out each instruction as I say it. When I have finished reading out the instructions I want you to act out everything I just said, in the same order. So if I read out ‘lift the square, push the circle’, you would act out ‘lift the square’ as soon as I say it, then you would act out ‘push the circle’ as soon as I say it. When I’ve finished reading the instructions, you would then act out ‘lift the square, push the circle’ again. We’re going to do some practicing first, so you can get used to what you need to do. In the practice, we will start with one thing and then there will be more for you to remember each time.

### Experiment 2a

#### No demonstration at encoding/verbal recall

Instructions were exactly the same as in Experiment 1

#### No demonstration at encoding/enacted recall

Instructions were exactly the same as in Experiment 1

#### Demonstration at encoding/verbal recall

Now I am going to read out the instructions, and you have to watch me as I act them out. When I have finished reading out the instructions I want you to tell me everything I just said, in the same order. So if I read out ‘lift the square, push the circle’, you would watch me act out ‘lift the square, push the circle’ as I say it. When I’ve finished reading the instructions, you would then say ‘lift the square, push the circle’. We’re going to do some practicing first, so you can get used to what you need to do. In the practice, we will start with one thing and then there will be more for you to remember each time.

#### Demonstration at encoding/enacted recall

Now I am going to read out the instructions, and you have to watch me as I act them out. When I have finished reading out the instructions, I want you to act out everything I just said, in the same order. So if I read out ‘lift the square, push the circle’, you would watch me act out ‘lift the square, push the circle’ as I say it. When I’ve finished reading the instructions, you act out ‘lift the square, push the circle’. We’re going to do some practicing first, so you can get used to what you need to do. In the practice, we will start with one thing and then there will be more for you to remember each time.

## Footnotes

1 Other than replacing the object in its original place, the experimenter did not interact with the objects during the test phase

2 An ANOVA was also run with age as an IV (grouped by school year). Overall, the main effect of age was significant, with older children performing better on the task than younger children. However, importantly, there were no interactions between age and encoding, or age and recall. Children across all ages showed the same pattern of responses across the different encoding and recall conditions.

3 An earlier study had been conducted with 96 participants ages 6- to 10- years using this paradigm. This is not reported in detail here as the experimental task used a span methodology which produced a lack of variability in task scores, and it did not include additional working memory measures. However, the results showed the same pattern with a significant positive effect of enactment at recall, and a significant negative effect of enactment at encoding.

4 As with Experiment 1, a three-way ANOVA was run with age as an IV (grouped by school year). There was a significant main effect of age, with the older children performing better on the task overall. However, as with Experiment 1, the pattern of responses across different year groups was the same across the different conditions.
